# Unveiling the Role of CB2 Receptor in Beta-Hydroxybutyrate Mediated Modulation of Microglial Anti-inflammatory Phenotype

**DOI:** 10.1007/s13105-026-01206-x

**Published:** 2026-07-09

**Authors:** Monika Iešmantaitė, Liutauras Usonis, Kristupas Čeika, Egidijus Šimoliūnas, Mantas Liudvinaitis, Nerea Soto-Arroyo, Isabel Moreno-Indias, Francisco J Tinahones, Daiva Baltriukienė, Virginia Mela

**Affiliations:** 1 Institute of Biochemistry, Life Sciences Center, Vilnius University, Vilnius, Lithuania; 2 Department of Endocrinology and Nutrition, Instituto de Investigación Biomédica de Málaga y Plataforma en Nanomedicina–IBIMA Plataforma Bionand, Málaga, Spain; 3 Center for Biomedical Network Research in Physiopathology of Obesity and Nutrition (CIBEROBN), Instituto de Salud Carlos III, Madrid, Spain; 4 Department of Endocrinology and Nutrition, Hospital Universitario Virgen de la Victoria–IBIMA Plataforma Bionand, Málaga, Spain; 5 Department of Medicine and Dermatology, Facultad de Medicina, Universidad de Málaga, Málaga, Spain; 6 Department of Surgical specialties, biochemistry, and immunology, Universidad de Malaga, Campus de Teatinos s/n, Malaga, Málaga, 29010 Spain; 7 Department of Biological Models, Vilnius University Life Sciences Center, Saulėtekio av. 7, Vilnius, 10257 Lithuania

**Keywords:** Neuroinflammation, Endocannabinoid system, Cannabinoid receptor 2, Ketone bodies, β-Hydroxybutyrate

## Abstract

The cannabinoid receptor type 2 (CB2R), primarily expressed in microglia, the brain’s resident immune cells, acts as a central regulator of neuroinflammatory responses. When CB2R is activated, it triggers anti-inflammatory signaling, making it a promising target for modulating microglial function in neuroinflammatory diseases. The ketone body, β-hydroxybutyrate (BHB), is gaining attention as a therapeutic agent for neurodegenerative disorders due to its ability to modulate neuroinflammation and preserve blood–brain barrier integrity. One mechanism by which BHB exerts anti-inflammatory effects is through regulation of microglial function; however, the precise mechanisms remain unclear. Since the role of BHB in this context is unexplored, we used two neuroinflammation models to test the hypothesis that CB2R-associated signaling contributes to the effects of BHB. In a mouse model of diet-induced obesity (DIO), characterized by chronic low-grade neuroinflammation, BHB treatment promoted ramified microglial morphology and enhanced debris clearance while sparing synaptic elements. These changes were accompanied by alterations in CB2R-related signaling markers and a slight increase in hydroxycarboxylic acid receptor 2 (HCA2), a known BHB target. When primary microglial cultures were challenged with lipopolysaccharide (LPS), BHB helped restore their function. However, that benefit disappeared when CB2R was pharmacologically blocked. Importantly, BHB increased the expression of arginase 1 (Arg1), a hallmark of anti-inflammatory responses, a change reversed by CB2R blockade. Moreover, BHB reduced NF-κB signaling, and CB2R inhibition attenuated this effect, suggesting that CB2R-associated signaling contributes to BHB’s anti-inflammatory actions. Collectively, our findings demonstrate that BHB’s anti-inflammatory effects are mediated, at least in part, through CB2R signaling, providing new insight into its therapeutic potential for neuroinflammation.

**Supplementary Information:**

The online version contains supplementary material available at https://doi.org/10.1007/s13105-026-01206-x.

## Key points

• BHB supplementation attenuated DIO-associated microglial activation and reduced selected inflammatory markers in the brain.

• In primary microglia, BHB reduced LPS-induced inflammatory responses and modulated microglial functional activity.

• Pharmacological blockade of CB2R partially reversed selected BHB-induced effects, suggesting involvement of CB2R-associated signaling.

• The findings support an indirect modulatory role of CB2R in BHB-mediated anti-inflammatory effects.

**Supplementary Information:**

The online version contains supplementary material available at https://doi.org/10.1007/s13105-026-01206-x.

## Introduction

Neuroinflammation is a complex response within the central nervous system (CNS) to stimuli such as trauma, infection, or neurodegenerative diseases [1]. While its primary function is to protect the brain, prolonged or dysregulated neuroinflammation can become detrimental to neural tissue [2]. Chronic neuroinflammation is characterized by persistent overactivation of microglia, leading to the release of inflammatory mediators that increase oxidative stress [3]. Microglia are the primary immune cells of the central nervous system (CNS) and play critical roles in both defense and neuropathology, depending on the environment [2]. Upon overstimulation, microglia transform from a ramified to an amoeboid morphology [4], a process accompanied by increased expression of pro-inflammatory cytokines such as interleukin (IL)-1β, IL-6, and tumor necrosis factor-α (TNF-α), which affect the surrounding brain tissue [5]. If this activation persists and becomes chronic, microglial function is impaired [6]. This perpetuates a damaging cycle of inflammation, contributing to neuronal loss. Therefore, many researchers are focused on targeting microglial cells as a powerful mechanism to combat neurodegenerative diseases [7]. Since there is no effective treatment for neurodegenerative diseases yet, clinicians focus their efforts of promoting healthier lifestyle habits [8–10]. Nowadays, dietary regimens are receiving increasing attention as a means to combat various diseases or to at least alleviate their symptoms. One of the most recently studied diets is the ketogenic diet, which has pathophysiological and clinical benefits in neurodegenerative diseases [11] and improves cognitive function [12]. Recent evidence has highlighted the anti-inflammatory potential of β-hydroxybutyrate (BHB), the main ketone body produced during states of ketosis induced by this diet [13–15]. BHB alone has been shown to be helpful in various experimental models of neurodegenerative diseases [16, 17], suggesting that it may have therapeutic benefits in neuroinflammatory conditions. BHB clearly exerts its anti-inflammatory effects through the modulation of microglia activity. Although some studies have shown that BHB affects various pathways through HCA2, such as the NLRP3 inflammasome and the NF-κB signaling pathway [18, 19], in microglia, the precise mechanisms by which BHB modulates microglial activity remain unclear.

The endocannabinoid system is gaining increasing attention in the context of neurodegenerative disorders and neuroinflammation, as evidence suggests that synthetic and endogenously produced cannabinoids can reduce microglial pro-inflammatory responses and promote a switch to an anti-inflammatory phenotype [20–21]. This system comprises cannabinoid receptors, endogenous cannabinoids, and enzymes that synthesize and metabolize endocannabinoids [22]. Among its receptors, cannabinoid receptor type 2 (CB2R) is predominantly expressed in immune cells and, to a high degree, in microglia, where it plays a modulatory role in inflammation and microglial activation [23]. Indeed, its activation has been shown to attenuate pro-inflammatory signaling [24]. During inflammatory events, the brain can produce endocannabinoids, most notably anandamide and 2-arachidonylglycerol, which exert anti-inflammatory effects by activating CB2R [25]. Although some synthetic cannabinoid receptor agonists are used [26–28], most are associated with adverse effects [29]. Therefore, it remains unclear whether there are compounds that can indirectly activate the anti-inflammatory cascade, without directly binding to the cannabinoid receptors, thereby achieving beneficial effects without the associated side effects.

In this study, we hypothesize that BHB exerts its anti-inflammatory effects in the brain by activating CB2R in microglia. Using a mouse model of diet-induced obesity (DIO), which is considered a model of mild inflammation [30–32], we assessed microglial state and function as well as CB2R, MAGL, and HCA2 expression in the hippocampi of mice, a brain region susceptible to dietary factors and obesity [33–35]. To further explore the mechanistic role of CB2R, we conducted in vitro experiments using primary microglial cultures stimulated with LPS to mimic an inflammatory environment and evaluated the effects of BHB and CB2R receptor antagonism on microglial phagocytosis, migration, ROS production, expression of pro-inflammatory genes, and NF-κB signaling. These findings provide insights into the interaction between BHB and CB2R signaling in modulating microglial function and offer potential therapeutic avenues for targeting neuroinflammation.

## Methods

### Animal studies

Eight-week-old male C57BL/6 mice were used for all experimental procedures. All animal procedures were approved by the Ethics Committee for Animal Experimentation of the University of Málaga (CEUMA) and authorized by the competent authority of the Andalusian Regional Government (protocol no. 04/05/2021/064). All procedures were conducted in accordance with Spanish legislation (Royal Decree 53/2013) and Directive 2010/63/EU on the protection of animals used for scientific purposes. Mice were housed in groups of five per cage under standardized conditions (20–22 °C), with free access to food and water, and monitored under veterinary supervision. No adverse effects were reported. To reduce experimental bias, mice were randomly assigned to treatment groups.

### Diet-induced obesity (DIO) mouse model and ketone body supplementation

**Fig. 1:**
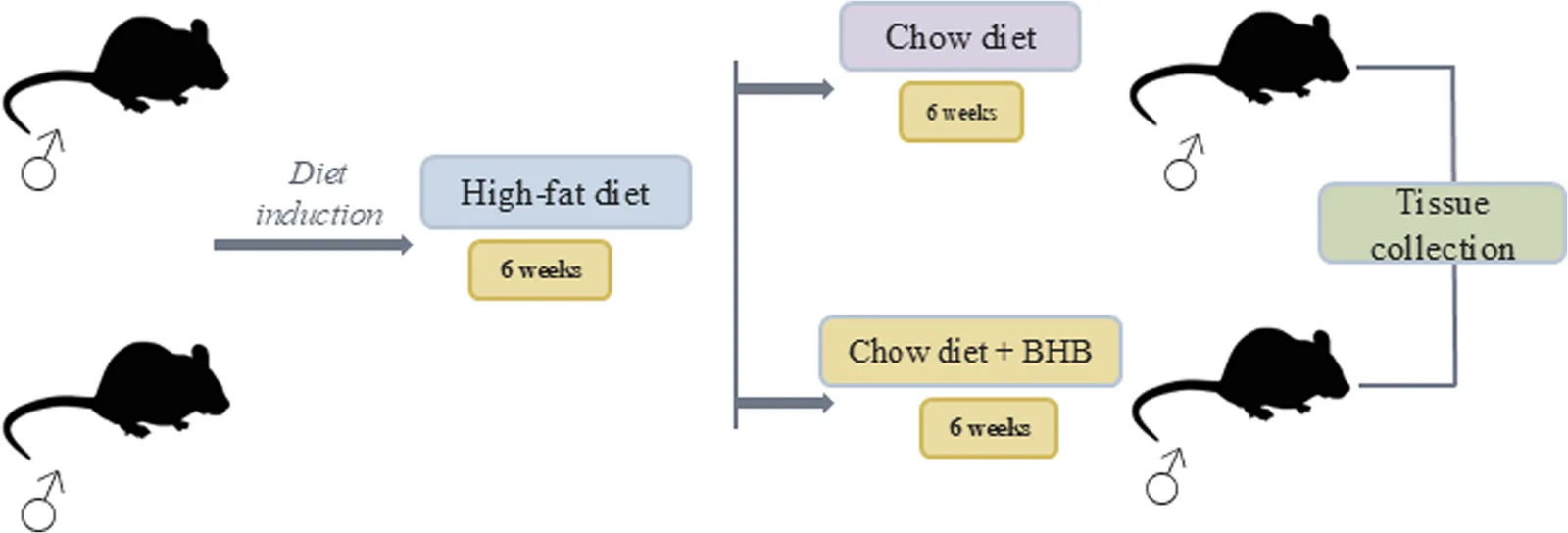
Experimental timeline of the DIO mouse model and BHB treatment. Male mice were fed a high-fat diet for 6 weeks to induce diet-induced obesity. After this induction period, mice were divided into two groups and maintained for an additional 6 weeks on either a chow diet or a chow diet supplemented with BHB. At the end of the treatment period, brain tissue was collected for further analysis

The DIO model and ketone body supplementation were performed as previously described [36]. In brief, 20 mice were fed a 60% high-fat diet (HFD; Brogaarden ApS) for six weeks to induce an obese phenotype, defined by a 20–30% body weight gain [37, 38]. Following this period, a chow diet was introduced. To supplement ketone bodies, half of the animals (BHB group) were administered BHB (KetoForce food supplement) at a concentration of 163.8 g/L in the drinking water for six weeks, while the remaining mice (Con group) received regular drinking water as a control, as previously reported at [36] (Fig. 1). Fresh BHB suspensions were prepared daily to prevent the substance from precipitating.

### Immunohistochemistry

The brain was dissected, and the tissue was fixed in 4% paraformaldehyde (PFA) for 24 h. Following fixation, the tissue was incubated in 30% sucrose for 48 h for cryoprotection and subsequently frozen in isopentane. The samples were embedded in optimal cutting temperature (OCT) compound, and 20 μm coronal sections were prepared using a cryostat. Sections were stored at − 20 °C in a freezing solution (30% ethylene glycol, 30% sucrose in PBS) until immunohistochemical processing. Immunohistochemistry was performed on brain slices containing the medial hippocampus. The sections were washed, permeabilized in PBT (PBS + 0.3% Triton X-100), and blocked for 1 h in PBT containing 3% bovine serum albumin (BSA). The samples were then incubated overnight at 4 °C with primary antibodies under continuous rocking motion: CD68 (Abcam, #AB303565, Abcam, 1:350), PSD-95 (SYSY antibodies, #124 011, 1:250), Iba-1 (WAKO, #019-19741, 1:500). After washing, sections were incubated for 2 h at room temperature with secondary antibodies at a dilution of 1:1000 in blocking buffer: Alexa Fluor 568 (Invitrogen, #A-21124), Alexa Fluor 488 (Invitrogen, #A-11008). Nuclei were counterstained with DAPI (1:8000, ThermoFisher) for 10 min at room temperature. Brain sections were mounted in ProLong^®^ Glass Antifade Mountant (P36984, ThermoScientific). Images were acquired using a Leica SP8 Stellaris scanning confocal microscope (3 fields per section, 40× magnification for morphology analysis and CD68/Iba-1 colocalization, 63× magnification for PSD95 phagocytosis), and processed using ImageJ software (National Institute of Health, https://imagej.org). To assess PSD95 engulfment by Iba-1⁺ cells, 3D reconstruction and analysis were performed using IMARIS software (Bitplane, Oxford Instruments).

### Morphological analysis

Immunostaining of the microglial marker Iba-1 was used to evaluate whether morphological alterations occur in microglia from the Con and BHB-fed mice. Image stacks (0.5 μm step size; 33 steps) were taken from Iba1-stained hippocampal tissue Sect.  (20 μm) using a Leica SP8 Stellaris scanning confocal microscope. Image analysis was performed using ImageJ. Images were converted to 8-bit greyscale, a threshold was set, and binarised images were filtered by pixel size to reduce background and enhance contrast. A skeleton analysis was used to assess changes in features relevant to microglia ramification, whereas fractal analysis was used to evaluate characteristics associated with cell surface, soma thickness, cell size, the complexity of their ramifications, and the heterogeneity of their shape.

### Enzyme-linked immunosorbent assay (ELISA)

IL-6 concentration was measured in homogenized cortical tissue from both treatment groups using a Mouse IL-6 ELISA kit (Duo-set R&D) according to the manufacturer’s instructions. Briefly, 96-well plates were coated with goat anti-mouse IL-6 capture antibody (R&D Systems, US), incubated overnight at room temperature, and blocked with 1% BSA in PBS for 1 h at room temperature. Duplicate samples and standards were added and incubated for 2 h at room temperature. Plates were washed and incubated with biotinylated goat anti-mouse IL-6 detection antibody for 2 h at room temperature. Plates were washed and incubated with HRP-conjugated streptavidin for 20 min, and then washed again. Substrate solution (1:1 H2O2:tetramethylbenzidine; Sigma-Aldrich, UK) was applied and incubated for 20 min at room temperature in the dark. The reaction was stopped with 1 M H2SO4, and absorbance was measured at 450 nm using a BioTek Agilent microplate reader (Agilent Technologies). The concentrations were calculated from the calibrated standard curve.

### Western blot analysis

Total protein was extracted from the organic phase of the RNA isolation protocol using TRIzol™ Reagent (ThermoFisher Scientific) according to the manufacturer’s protocol. The protein concentration in the supernatant was determined using PierceTM BCA protein assay (ThermoFisher Scientific; Cat# 23225). Proteins were denatured in Laemmli buffer containing 5% 2-β-mercaptoethanol at 95 °C for 5 min. 20 µg of protein per sample was loaded onto 12% acrylamide gels and subjected to SDS-PAGE. Proteins were then transferred to a PVDF membrane and blocked in 5% non-fat dried milk powder in Tris-Tween buffered saline (TTBS) for 1 h. Primary antibodies were diluted in TTBS with 5% non-fat dried milk powder as follows: Arg1 (Invitrogen, PA5-85267, 1:500), CB2R (Invitrogen, #703485, 1:500), MAGL (Abcam, #ab24701, 1:100), IL-1β (PeproTech, 500-P51-1MG, 1:750), HCA2 (Invitrogen; #MA5-51337) α-tubulin (Invitrogen, # PA5-85922, 1:5000). Membranes were incubated with antibodies overnight at 4 °C at constant rotation. After washing in TTBS (3 × 10 min), membranes were incubated at room temperature for 2 h in horseradish peroxidase-labeled goat anti-rabbit secondary antibodies (Jackson ImmunoResearch, #111-035-003, 1:2000, Glendora, CA, USA) in 5% non-fat dried milk powder in TTBS. After washing (3 × 10 min), membranes were treated for 5 min with ClarityTM Western ECL Substrate (Bio-Rad, #170–5061). Protein band intensities were captured using the ChemiDoc™ MP digital imager (Bio-Rad Laboratories, Hercules, CA, USA).

Image Lab software (Bio-Rad Laboratories, Hercules, CA, USA) was used for image analysis to determine signal intensities of identified bands. The bands of interest were then normalized to the tubulin intensity of a given lane.

### Preparation of L929 conditioned medium

The L929 cell line (ATCC, CCL-1™) was cultured in DMEM/F12 medium supplemented with 10% heat-inactivated fetal bovine serum (HI FBS) and 1% penicillin-streptomycin solution (10,000 units/mL penicillin and 10,000 µg/mL streptomycin) at 37 °C in a humidified atmosphere of 5% CO2. The L929 cells were grown in T-75 cell culture flasks and allowed to reach 90% confluency. The culture medium was then discarded and replaced with 30 mL of fresh medium, and the conditioned medium was collected 14 days later. The supernatant was centrifuged at 300 × g for 10 min, filtered using 0.22 μm syringe filters, aliquoted into sterile tubes, and stored at − 80 °C until use.

### Mouse microglia isolation and culture

Microglia were isolated as described by Scott et al. [39] with minor modifications. Briefly, C57BL/6J neonatal (P0-P3) mice were culled, brains were extracted, and the meninges were removed. The brain tissue was digested in 30 mL of dissection medium (HBSS with Ca^2+^ and Mg^2+^, 4.5 g/L glucose, 10 mM HEPES, and 1× Antibiotic-Antimycotic solution) supplemented with 2.5% trypsin/EDTA solution at 37 °C for 15 min. Afterward, 1.5 mL of trypsin inhibitor (1 mg/mL) was added, followed by 750 µL of DNase I (10 mg/mL) to stop the reaction. The mixture was centrifuged at 400 × g for 5 min at 20 °C. The cell pellet was resuspended in 5 mL of warm culture medium (DMEM/F-12 supplemented with 10% HI FBS and 1% penicillin/streptomycin solution), centrifuged again under the same conditions, and the pellet was resuspended in 10 mL of fresh medium and transferred to T-75 flasks pre-coated with 0.1 mg/mL poly-L-lysine (Sigma Aldrich).

The next day, the medium was replaced with fresh culture medium supplemented with 10% L929-conditioned medium, and the flasks were incubated for an additional 13 days. Microglia were isolated from the culture medium by centrifugation at 800 × g for 5 min. The cell pellet was resuspended in fresh culture medium, counted, and seeded for further experiments. For confocal microscopy, 50,000 cells were seeded onto 13-mm glass coverslips in 24-well plates. For RNA and protein extraction, 500,000 microglia cells were seeded into 6-well plates.

### Cell treatments

On the first day, cells were seeded at an appropriate density, as mentioned above. On the following day, cells were treated under the following conditions: lipopolysaccharide (LPS) (Sigma Aldrich) alone (100 ng/mL) for 24 h; BHB (Sigma Aldrich) (10 mM) for 1 h, followed by LPS (100 ng/mL) for 24 h or AM-630 (Sigma Aldrich) (1 µM) for 1 h, followed by BHB (10 mM) for 1 h, and then LPS (100 ng/mL) for 24 h.

### Real-time qPCR analysis

Total RNA was isolated using TRIzol™ Reagent (ThermoFisher Scientific) according to the manufacturer’s protocol. The RNA yield and quality were quantified using the Nanodrop spectrophotometer (NanoPhotometer N60/N50). cDNA synthesis was performed using the High-Capacity cDNA Reverse Transcription Kit (Applied Biosystems) following the manufacturer’s instructions and using the maximum amount of RNA per sample.

For in vivo samples, real-time PCR was performed on duplicate samples using KAPA Probe Fast (VWR, KK4702) with predesigned TaqMan gene expression assays for IL-6 (Mm00446190_m1), IL-1β (Mm00434228_m1), and TNF-α (Mm00433832_m1) on a CFX 96 Real-Time System. Samples were assayed with β-actin (VIC/MGB probe, primer limited, 4 352 341E, ThermoFisher) as the endogenous control to normalize gene expression data.

For in vitro samples, QPCR was performed using the PowerUp™ SYBR™ Green Master Mix according to the manufacturer’s protocol. Each reaction was performed in duplicate. The primers used for gene expression analysis are listed in Table 1. QPCR was carried out in QuantStudio™ 5 Real-Time PCR System (ThermoFisher Scientific). Gene expression data were analyzed using the ΔΔCt method and normalized to the housekeeping gene glyceraldehyde-3-phosphate dehydrogenase (*GAPDH*), which served as an endogenous control for quantification assays.

**Table 1:** Sequences of specific primers used for quantitative real-time PCR

Gene	Forward primer sequence 5’→3’	Reverse primer sequence 5’→3’
*TNF-α*	ATGGCCTCCCTCTCATCAGT	TTTGCTACGACGTGGGCTAC
*Arg1*	CTTGCGAGACGTAGACCCTG	TCCATCACCTTGCCAATCCC
*IL-10*	CGGGAAGACAATAACTGCACCC	CGGTTAGCAGTATGTTGTCCAGC
*GAPDH*	TGTCGTGGAGTCTACTGGTGTCTTC	CGTGGTTCACACCCATCACAA
*NOX-2*	CGAAGACAACTGGAAGGAA	GCTCVCACTAACATCACCAC
*TGF-β*	ACTGGAGTTGTACGGCAGTG	GGGGCTGATCCCGTTGATTT

### Phagocytosis assay

Following treatment, 0.025% latex beads (1 µm^2^, carboxylate-modified polystyrene, fluorescent yellow-green, Sigma-Aldrich) were added to the culture medium, and the cells were incubated at 37 °C, 5% CO_2_ for 4 h. After incubation, microglia were fixed with 4% PFA for 15 min, blocked with 3% BSA and 10% FBS in PBS for 1 h, and then incubated with an anti-CD11b-PE antibody (Invitrogen, #12-0112-82, dilution 1:150) to visualize their morphology. Nuclei were counterstained with DAPI (1 µg/mL) (ThermoFisher Scientific) for 5 min. Coverslips were mounted onto microscopy slides using Moviol mounting medium, and samples were stored in the dark at room temperature for at least 24 h. Cells were visualized using a Leica SP8 Stellaris scanning confocal microscope (3 fields; 63× magnification). To quantify phagocytosis, the ImageJ Z-Project function was used to process the images, and the number of beads per cell was manually counted.

### Assessment of the production of ROS

ROS generation was assessed using CellROX™ Green reagent (ThermoFisher Scientific) according to the manufacturer’s instructions. Briefly, CellROX solution was added to the culture medium at a final concentration of 5 µM following treatment, and cells were incubated in the dark for 30 min at 37 °C, and 5% CO_2_. After incubation, cells were fixed with 4% PFA for 15 min. Nuclei were counterstained with DAPI (1 µg/mL) for 5 min. Coverslips were mounted onto microscopy slides using Moviol mounting medium, and samples were stored at room temperature in the dark for at least 24 h. Cells were visualized using a Leica SP8 Stellaris scanning confocal microscope (3 fields; 20X magnification). Analysis was performed using ImageJ software. Background fluorescence was subtracted, and the fluorescence intensity of individual cells was measured using the Integrated Density function.

### NF-κB localization analysis

Cells were fixed and permeabilized as previously described. Immunolabeling was performed using a primary antibody against NF-κB (Invitrogen, #14-6731-63, 1:100), followed by incubation with a secondary antibody AlexaFlour 488 (Invitrogen, #A11008, 1:1000). Nuclei were counterstained with DAPI (1 µg/mL) (ThermoFisher Scientific) for 5 min. Coverslips were mounted onto microscope slides as described above. Image analysis was conducted using ImageJ software. First, regions of interest (ROIs) were selected for the whole cell, and both mean fluorescence intensity and area were measured. Subsequently, nuclear ROIs were defined based on the DAPI channel and corresponding NF-κB signal, and their mean intensity and area were calculated. Cytoplasmic NF-κB intensity (cyt.int.) was determined using the following formula (1):

1 $$\:Cyt.\:int.=\left(cell\:mean\:int.*cell\:area\right)-\left(nuclear\:mean\:int.*nuclear\:area\right)$$

Then, the cytoplasmic area was calculated using formula (2):

2 $$\:Cytoplasmic\:area=whole\:cell\:area-nuclear\:area$$

Cytoplasmic mean intensity was calculated following formula (3):

3 $$\:Cytoplasmic\:mean\:int.=\frac{cytoplasmic\:intensity}{cytoplasmic\:area}$$

Finally, nuclear/cytoplasmic NF-κB intensity was calculated by dividing nuclear mean intensity by cytoplasmic mean intensity, and data were expressed as arbitrary units.

### Statistical analysis

All statistical analyses were performed using GraphPad Prism 10. Data are presented as mean ± SEM, with the number of biological replicates or experimental repeats indicated in each figure. Normality and homogeneity of variances were assessed using the Shapiro–Wilk and Levene’s tests, respectively. For comparisons between two independent groups, Student’s t-test was applied if the data were normally distributed and the variances were equal; Welch’s t-test was used when normality was met, but variances were unequal; and the Mann–Whitney U test was used if the data were not normally distributed. For comparisons involving more than two groups, one-way ANOVA followed by Tukey’s post hoc test was used when assumptions of normality and equal variances were met; and the Kruskal–Wallis test followed by Dunn’s test (with Bonferroni correction) was applied for non-parametric data. The significance level was set at *p* < 0.05.

## Results

### BHB treatment altered the morphology of microglia in the hippocampus of Obese mice

**Fig. 2:**
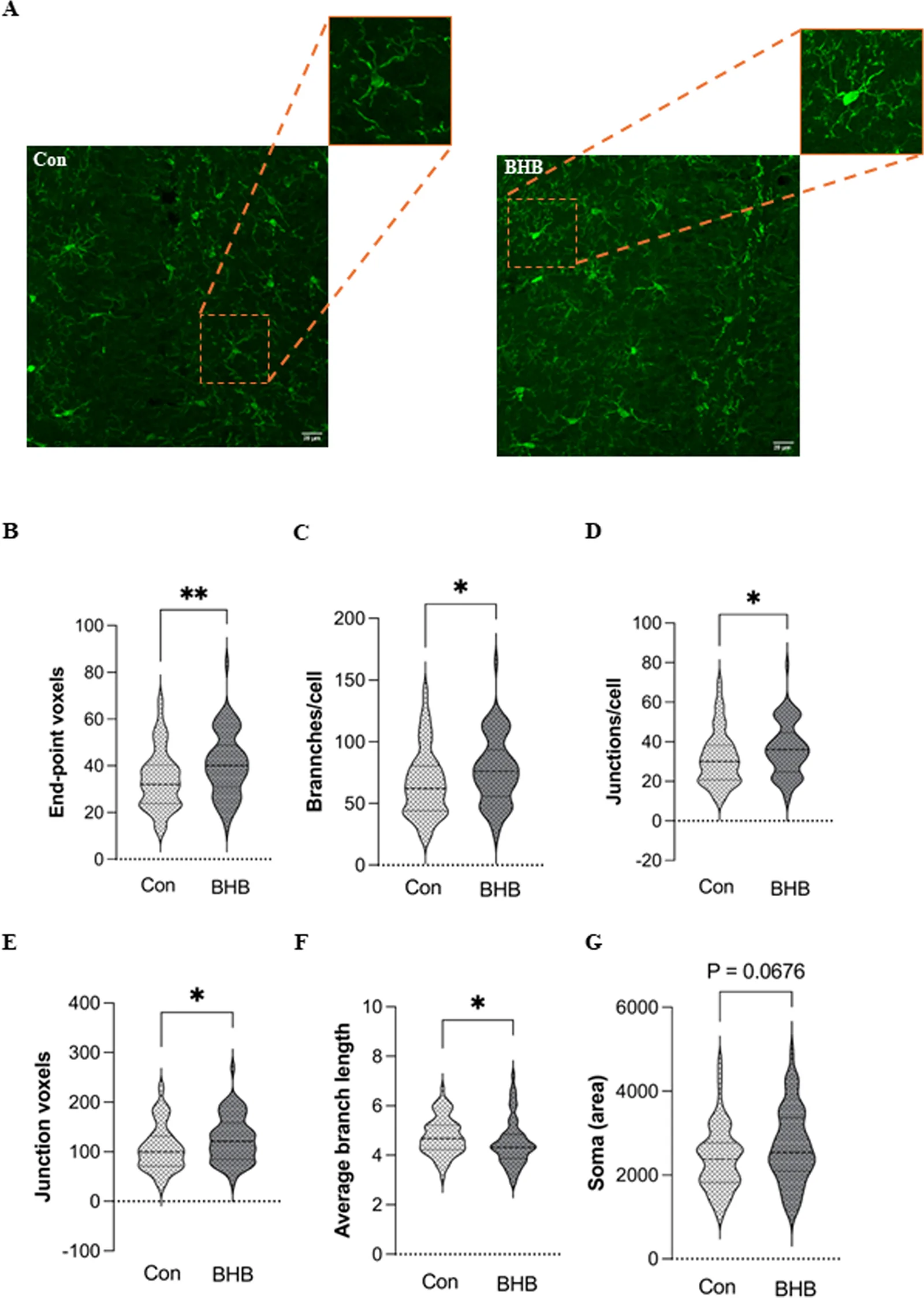
BHB treatment modified microglial morphology in the hippocampus of Obese mice. To assess the effect of BHB on microglial morphology in obesity-associated neuroinflammatory conditions, obese mice were treated with BHB, and hippocampal microglial morphology was analyzed by Iba-1 immunofluorescence and 3D morphological reconstruction. (**A**) Confocal microscopy images of microglia stained with Iba-1 antibody, showing microglia morphology; (**B**)# End-point voxels; (**C**) # Branches/cell; (**D**) # Junctions/cell; (**E**) # Junction voxels; (**F**) Average branch length; (**G**) Soma size. Con – control group, BHB – BHB treatment group. *n* = 6. For each animal, 10–15 microglial cells were analyzed. Data are presented as mean ± SEM; *p < 0.05; **p < 0.01

First, we aimed to assess changes in microglial complexity and branching patterns to investigate the potential effects of BHB treatment on microglial functional state in obese mice. To do this, we quantified morphological parameters in hippocampal microglia labeled with the Iba-1 marker (Fig. 2A). Analysis revealed significant differences in key structural parameters of microglia. Specifically, BHB-treated mice exhibited a notable increase in end-point voxels (Fig. 2B; *p* < 0.01), number of branches (Fig. 2C; *p* < 0.05), number of junctions (Fig. 2D; *p* < 0.05), junction voxels (Fig. 2E; *p* < 0.05), and a decrease in branch length (Fig. 2F; *p* < 0.05) compared to obese controls. Although the soma parameter did not reach statistical significance, there was a trend toward increased size (Fig. 2G; *p* = 0.068) in BHB-treated mice. These changes reflect increased microglial complexity and arborization, indicating that BHB may modulate microglial morphology in a manner consistent with a neuroprotective or anti-inflammatory phenotype in obese mice.

### BHB enhances microglial phagocytic activity and reduces synaptic engagement

To further characterize the effects of BHB on microglial state, we performed immunofluorescent colocalization analysis using CD68/Iba-1 and PSD95/Iba-1 labeling to evaluate microglial phagocytic functionality.

**Fig. 3:**
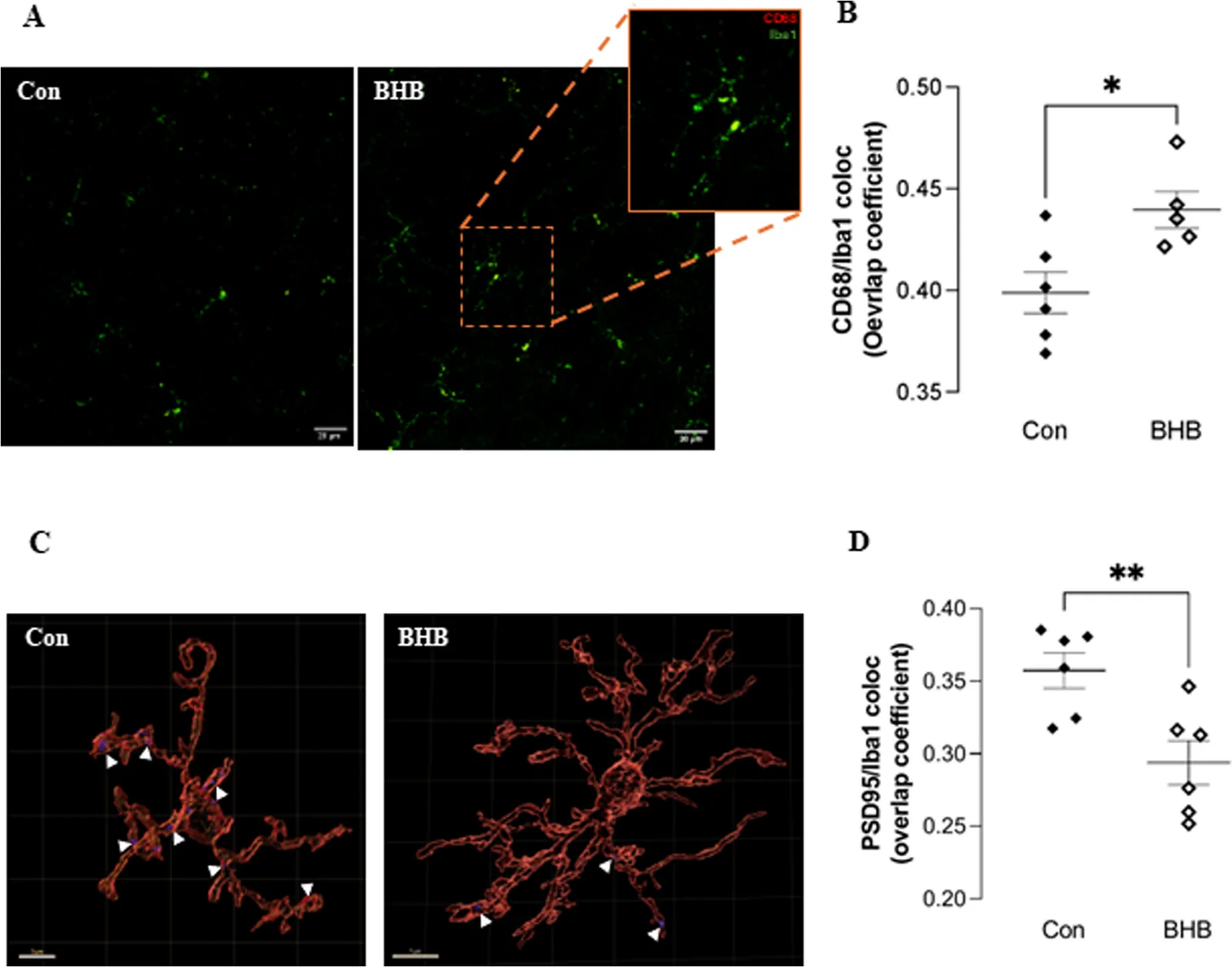
BHB treatment increases microglial phagocytic capacity while reduce synaptic pruning in the hippocampus of Obese mice (**A**) Representative confocal image showing CD68/Iba-1 colocalization in the hippocampus; (**B**) Quantification of CD68/Iba-1 overlap coefficient, showing increased colocalization in BHB-treated mice; (**C**) Representative IMARIS 3D reconstruction image showing PSD95 localization in microglia; (**D**) Quantification of PSD95/Iba-1 overlap coefficient, showing decreased colocalization in BHB-treated mice. Con – control group, BHB – BHB treatment group. *n* = 6. For each animal, 10–15 microglial cells were analyzed. Data are presented as mean ± SEM; *p < 0.05; **p < 0.01

Quantitative analysis of the overlap coefficient revealed a significant increase in CD68/Iba-1 colocalization in BHB-treated mice compared to controls (Fig. 3A-B; *p* < 0.05), indicating enhanced microglial phagocytic activity. Conversely, PSD95/Iba-1 colocalization was significantly reduced in the BHB group (Fig. 3C-D; *p* < 0.01), suggesting decreased microglial engulfment of postsynaptic elements.

### Neuroinflammation was reduced in the DIO mouse model by BHB treatment

**Fig. 4:**
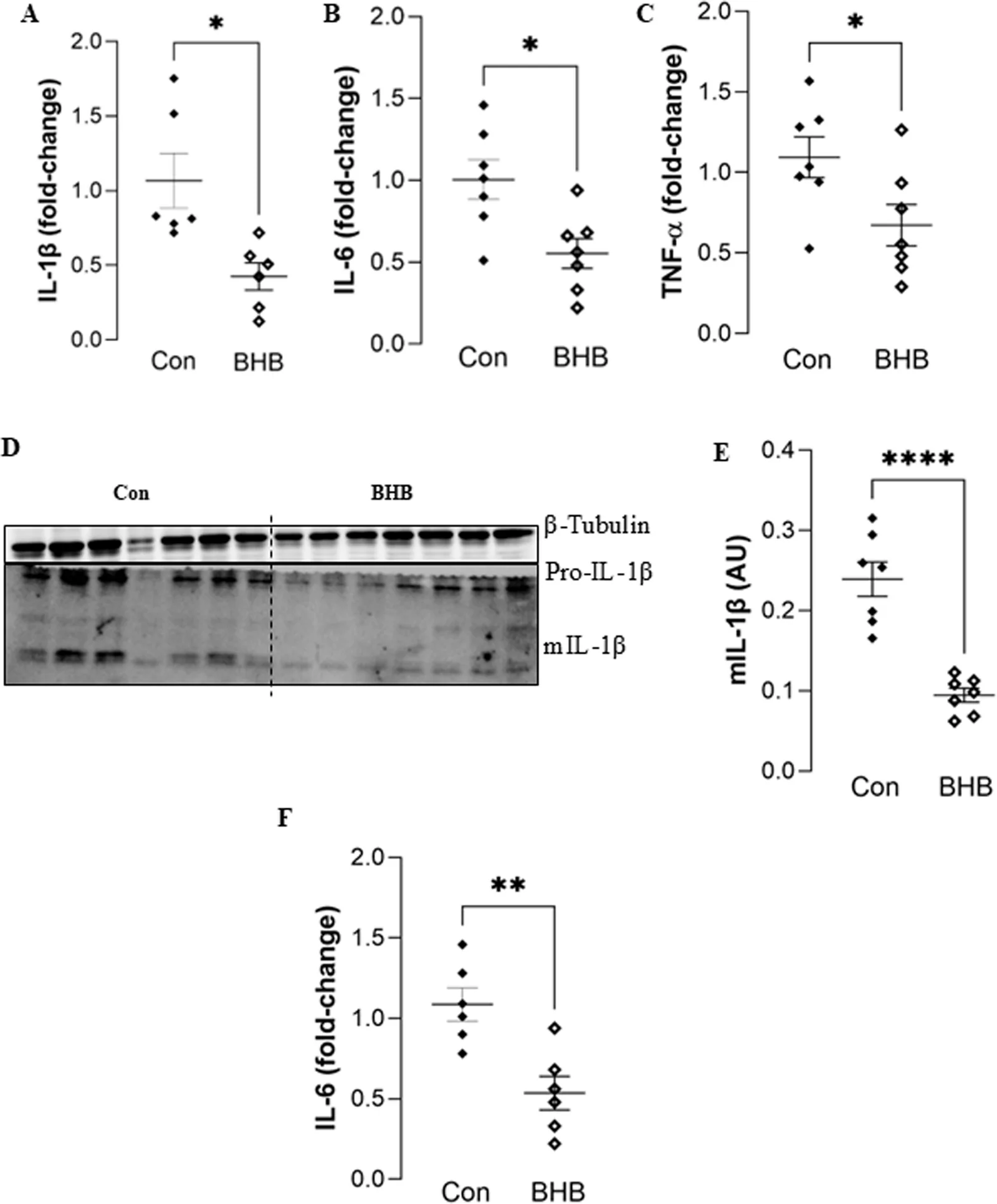
BHB treatment reduces pro-inflammatory cytokine levels in the brains of obese mice. **(A)** *IL-1β m*RNA expression fold-change compared to untreated control. **(B)** *IL-6* mRNA expression fold-change compared to untreated control. **(C)** *TNF-α* mRNA expression fold-change compared to untreated control. (**D**) Representative Western blotting data of cortex tissue from control and BHB-treated group showing pro-IL-1ß, mature IL-1ß (mIL-1ß), and α-tubulin expression. (**E**) Quantification of mIL-1ß expression by densitometric analysis normalized to α-tubulin. *n* = 7 (**F**) IL-6 expression measured by ELISA, *n* = 6–7. Con – control group, BHB – BHB treatment group. AU – arbitrary units. Data are presented as mean ± SEM; *p < 0.05; **p < 0.01; ****p < 0.0001

To assess the anti-inflammatory effects of BHB in the brains of obese mice, we measure mRNA expression of *IL-1β*,* IL-6*, and *TNF-α* in the hippocampus. BHB treatment significantly reduced the expression of all tested cytokines compared with untreated obese controls (Fig. 4. A, B, C; *p* < 0.05). To further evaluate the anti-inflammatory effects of BHB in the brain of obese mice, we measured IL-1β protein expression by Western blot and IL-6 levels by ELISA in cortical tissue, as mRNA expression does not always reflect protein abundance. BHB treatment significantly reduced IL-1β expression (Fig. 4D, E; *p* < 0.0001) and decreased IL-6 levels in the brain cortex compared to untreated obese controls (Fig. 4F; *p* < 0.01). These results indicate that BHB administration attenuates neuroinflammatory responses associated with obesity.

### CB2R, MAGL and HCA2 expression evaluation in the mouse hippocampi

**Fig. 5:**
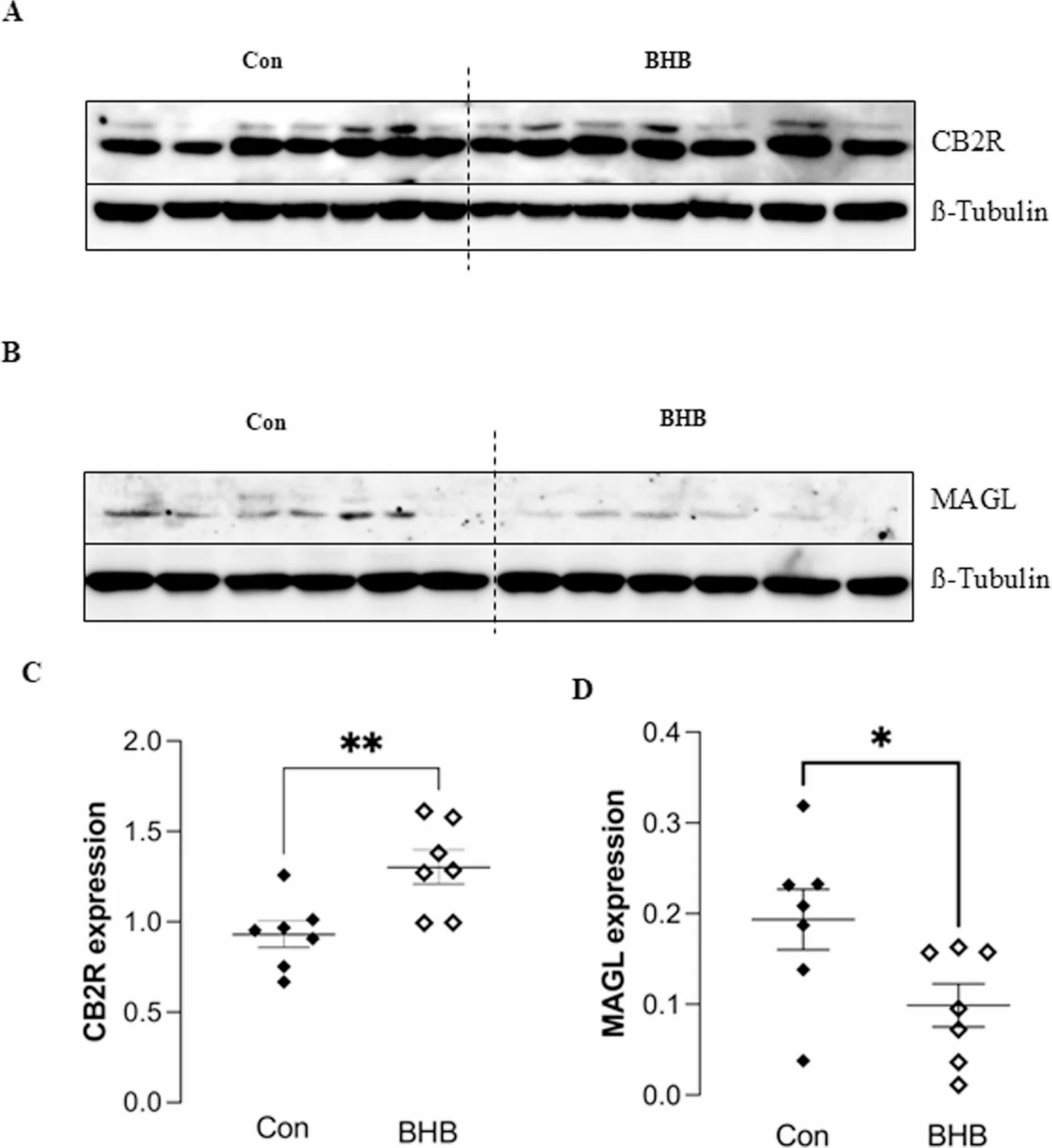
BHB modulates CB2R and MAGL expression in the hippocampus of obese mice **(A)** Representative Western blotting data of hippocampal tissue from control and BHB-treated groups showing CB2R and α-tubulin expression. (**B**) Representative Western blotting data of hippocampal tissue from control and BHB-treated groups showing MAGL and α-tubulin expression. (**C**) Quantification of CB2R expression by densitometric analysis normalized to α-tubulin. (**D**) Quantification of MAGL expression by densitometric analysis normalized to α-tubulin. Con – control group, BHB – BHB treatment group. *n* = 7. Data are presented as mean ± SEM. *p < 0.05; **p < 0.01

To investigate whether the endocannabinoid system contributes to the beneficial effects of BHB, we assessed the expression of CB2R, as well as MAGL – the primary enzyme responsible for endocannabinoid degradation – in both obese mice and those treated with BHB. We also evaluated hippocampal HCA2 expression, as HCA2 is an established receptor involved in BHB signaling. CB2R was detectable in hippocampal preparations from both control and BHB-treated mice. Notably, CB2R expression was significantly higher in the BHB-treated group compared to controls (Fig. 5A, C; *p* < 0.01). This finding suggests that BHB treatment is associated with altered CB2R expression in the hippocampus. Consistent with our hypothesis, MAGL expression was significantly reduced in BHB-treated mice (Fig. 5B, D; *p* < 0.05), suggesting a possible association between BHB administration and changes in components of the endocannabinoid system. HCA2 expression was higher in BHB-treated mice than in controls, although this difference did not reach statistical significance (Supplementary Fig. S1).

### CB2 receptor blockade suggests involvement of CB2 receptor-associated signaling in BHB-induced functional changes in primary microglia

To investigate whether BHB directly affects microglia, we performed in vitro experiments using primary microglial cells, focusing on phagocytic activity and ROS production, and examined whether these effects are influenced by CB2R-associated signaling.

**Fig. 6:**
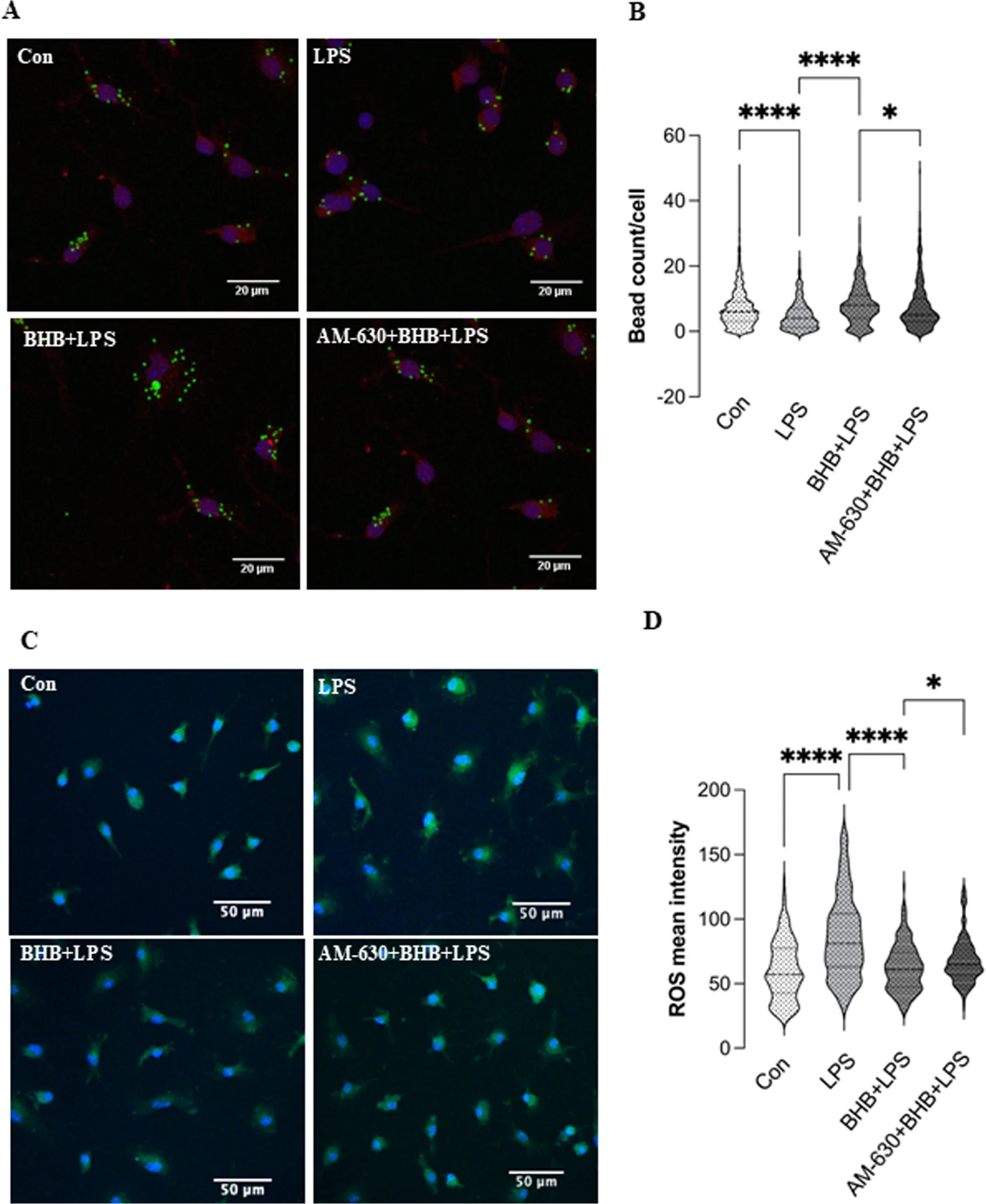
BHB promotes an anti-inflammatory microglial phenotype following inflammatory stimulation, with partial modulation by CB2R signaling (**A**) Representative fluorescence images showing phagocytosis of primary mouse microglia in different experimental groups. Green: internalized fluorescent latex beads; red: microglial marker CD11b; blue: nuclei stained with DAPI. (**B**) Violin plot depicting microglial phagocytosis of latex beads, *n* = 260–320. (**C**) Representative fluorescence images showing ROS production of primary mouse microglia in different experimental groups. Green: ROS signal; blue: nuclei stained with DAPI. (**D**) Violin plot depicting ROS synthesis, *n* = 200. Con – Control group; LPS – LPS-treated group (100 ng/mL); BHB + LPS – 1 h pretreatment with BHB before LPS; AM-630 + BHB + LPS – 1 h pretreatment with AM630 (1 µM) before BHB and LPS. Data are presented as mean ± SEM (three independent biological replicates). *p < 0.05; **p < 0.01; ****p < 0.0001

LPS treatment significantly impaired microglial phagocytosis, reducing the average number of internalized latex beads compared to the control group (Fig. 6A, B; *p* < 0.0001). Co-treatment with BHB and LPS significantly enhanced phagocytic activity relative to LPS alone (Fig. 6A, B; *p* < 0.0001), suggesting that BHB promotes microglial functional recovery under inflammatory conditions. Co-treatment with the CB2R antagonist AM-630, in the presence of BHB and LPS, resulted in a modest but statistically significant decrease in phagocytosis compared to the BHB + LPS group (Fig. 6A, B; *p* < 0.05). These results indicate that CB2R signaling contributes, at least in part, to the pro-phagocytic effects of BHB in microglia under inflammatory stress.

ROS levels were significantly elevated following LPS treatment (Fig. 6C, D; *p* < 0.0001), consistent with an activated pro-inflammatory microglial state. Pre-treatment with BHB significantly reduced ROS production compared to the LPS group (Fig. 6C, D; *p* < 0.0001), supporting its role in mitigating oxidative stress and exerting anti-inflammatory effects. Importantly, CB2R inhibition with AM-630 partially reversed the ROS-lowering effect of BHB, increasing ROS production relative to the BHB + LPS group (Fig. 6C, D; *p* < 0.05). These findings parallel the phagocytosis data and further suggest that CB2R blockade may promote a pro-inflammatory phenotype. Collectively, the results highlight the importance of CB2R signaling in mediating BHB’s anti-inflammatory and homeostatic effects in microglia.

### Regulation of pro- and anti- inflammatory gene expression in primary mouse microglia by BHB and the role of CB2R signaling

Next, we investigated whether BHB can downregulate pro-inflammatory gene expression in response to LPS stimulation, and whether this effect is mediated via the CB2R. To this end, we measured the mRNA expression levels of *TNF-α* and *NOX2* using qPCR.

**Fig. 7:**
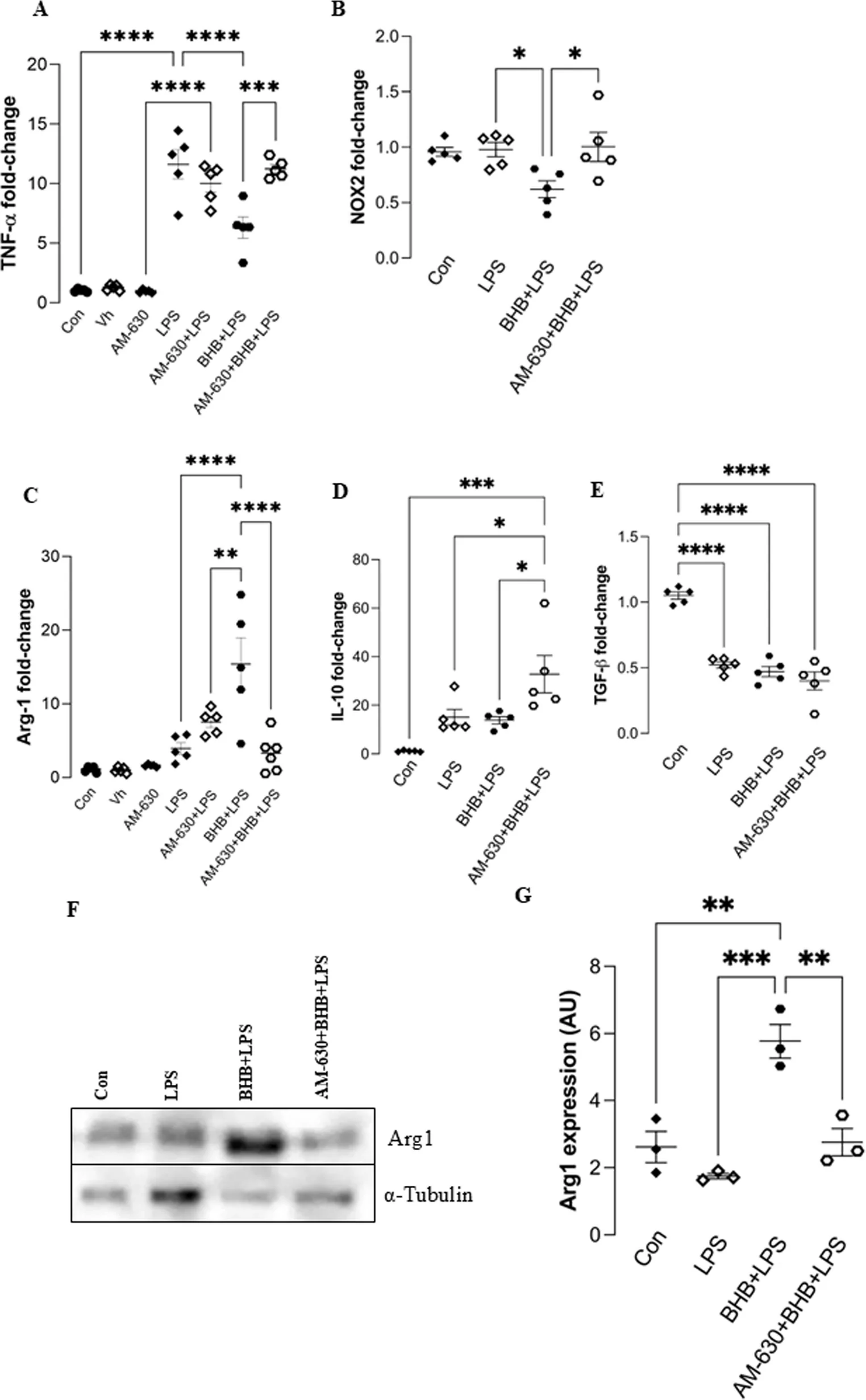
Modulation of pro- and anti-inflammatory cytokine expression in primary mouse microglial cells under LPS, LPS + BHB, and LPS + BHB+AM-630 treatments. (**A**) *TNF-α* mRNA expression fold-change compared to untreated control. (**B**) *NOX2* mRNA expression fold-change compared to untreated control. (**C**) *Arg-1* mRNA expression fold-change compared to untreated control. (**D**) *TGF-β* mRNA expression fold-change compared to untreated control. (**E**) *Arg1* mRNA expression fold-change compared to untreated control. Data is provided as 2^−∆∆Ct^ with individual points, *n* = 5 biological replicates per experimental group. Targets are normalized to the geometric mean of the reference gene *GAPDH*. (**F**) Representative Western blot bands showing Arg1 and α-tubulin expression in primary microglia cells. (**G**) Quantification of Arg1 expression normalized to α-tubulin. Con – Control group; Vh – vehicle; AM-630 – AM-630 treated group; LPS – LPS-treated group for 24 h (100 ng/mL); AM-630 + LPS – 1 h pretreatment with AM-630 before LPS; BHB + LPS – 1 h pretreatment with BHB before LPS; AM-630 + BHB + LPS – 1 h pretreatment with AM630 (1 µM) before BHB and LPS. *p < 0.05; **p < 0.01; ***p < 0.001; ****p < 0.0001

First, we showed that neither the vehicle nor AM-630 alone affected *TNF-α* expression, indicating that neither treatment had an intrinsic effect on *TNF-α* mRNA expression. As expected, *TNF-α* mRNA expression was significantly upregulated in the LPS-treated group (Fig. 7A; *p* < 0.001). Pretreatment with AM-630 before LPS exposure did not significantly alter TNF-α expression compared to LPS alone. Pre-treatment with BHB statistically significantly reduced *TNF-α* mRNA expression (Fig. 7A; *p* < 0.0001). Interestingly, pre-treated cells with the CB2R antagonist AM-630 prior to BHB and LPS exposure showed an increase in TNF-α mRNA expression compared to the LPS-only group (Fig. 6A; *p* < 0.05) and to the BHB + LPS group (Fig. 7A; *p* < 0.01). These findings suggest that while BHB alone does not significantly modulate *TNF-α* mRNA expression under inflammatory conditions, the increase observed with CB2R blockade implies a role for CB2R signaling in regulating *TNF-α* production. Since we did not find any significant effects of Vh or AM-630 alone, subsequent analyses were restricted to the groups that directly addressed the main experimental question, thereby allowing a clearer interpretation of the treatment-related effects.

*NOX2* mRNA expression remained unchanged following LPS treatment compared to the control group (Fig. 7B). However, in the group pre-treated with BHB prior to LPS stimulation, *NOX2* mRNA expression was significantly reduced compared to the LPS-only group (Fig. 7B; *p* < 0.05). Furthermore, when cells were pre-treated with the CB2R antagonist AM-630 before BHB and LPS exposure, *NOX2* mRNA expression increased relative to the BHB + LPS group (Fig. 6B; *p* < 0.05). These findings are consistent with the previously observed reduction in ROS levels and support the role of *NOX2* as a key contributor to ROS production. The data suggest that BHB attenuates oxidative stress by downregulating NOX2 and that this effect is at least partially mediated through CB2R signaling.

Additionally, we aimed to determine whether BHB upregulates the expression of anti-inflammatory genes and whether these effects are mediated via CB2R. To investigate this, we assessed the expression levels of *Arg-1*,* IL-10*, and *TGF-β* using quantitative PCR.

Arg1 mRNA expression, a marker of an anti-inflammatory microglial phenotype, remained unchanged after vehicle or AM-630 treatment alone, indicating that neither treatment directly affected Arg1 expression. LPS exposure modestly increased Arg1 expression; however, this change was not statistically significant. Pretreatment with AM-630 before LPS exposure produced a further slight increase in Arg1 expression compared with LPS alone, although this effect also did not reach statistical significance (Fig. 7C). However, pre-treatment with BHB significantly enhanced *Arg1* mRNA expression compared to the LPS-only group (Fig. 7C; *p* < 0.01). This BHB-induced upregulation was completely reversed by CB2R blockade, with AM-630 reducing *Arg1* mRNA relative to the BHB + LPS group (Fig. 7C; *p* < 0.001). Consistent with the approach described for the inflammatory markers, groups treated with vehicle or AM-630 alone were excluded from subsequent focused analyses, as neither condition had a significant independent effect on the measured variable.

After cells were treated with LPS, *IL-10* mRNA expression increased, but the difference was not statistically significant. BHB treatment did not significantly affect *IL-10* mRNA expression following LPS stimulation. Interestingly, co-treatment with the CB2R antagonist AM-630 increased IL-10 mRNA expression compared with the BHB + LPS group (Fig. 7D; *p* < 0.05).

LPS treatment led to a significant reduction in *TGF-β* expression (Fig. 7E; *p* < 0.001), and this effect was comparable in the BHB + LPS and AM-630 + BHB + LPS groups. This indicates that the effect of LPS is not altered by BHB treatment or CB2R blockade, suggesting that neither BHB nor CB2R mediates anti-inflammatory effects through this pathway (Fig. 7E).

However, mRNA expression levels do not always correlate directly with protein abundance. Therefore, we next aimed to determine whether Arg1 protein levels reflected the changes observed at the transcriptional level following treatment.

Arg1 protein expression followed a pattern consistent with its known role as an anti-inflammatory marker. LPS treatment reduced Arg1 levels, although the difference was not statistically significant (Fig. 7F, G). Notably, co-treatment with BHB and LPS resulted in an increase of Arg1 protein expression compared to LPS alone (Fig. 7F, G; *p* < 0.001). This effect was significantly reversed by AM-630 (Fig. 7F, G; *p* < 0.01), further supporting a CB2-mediated mechanism.

### BHB’s anti-inflammatory effect is mediated through the NF-κB pathway, and it is blocked with CB2R blockade

**Fig. 8:**
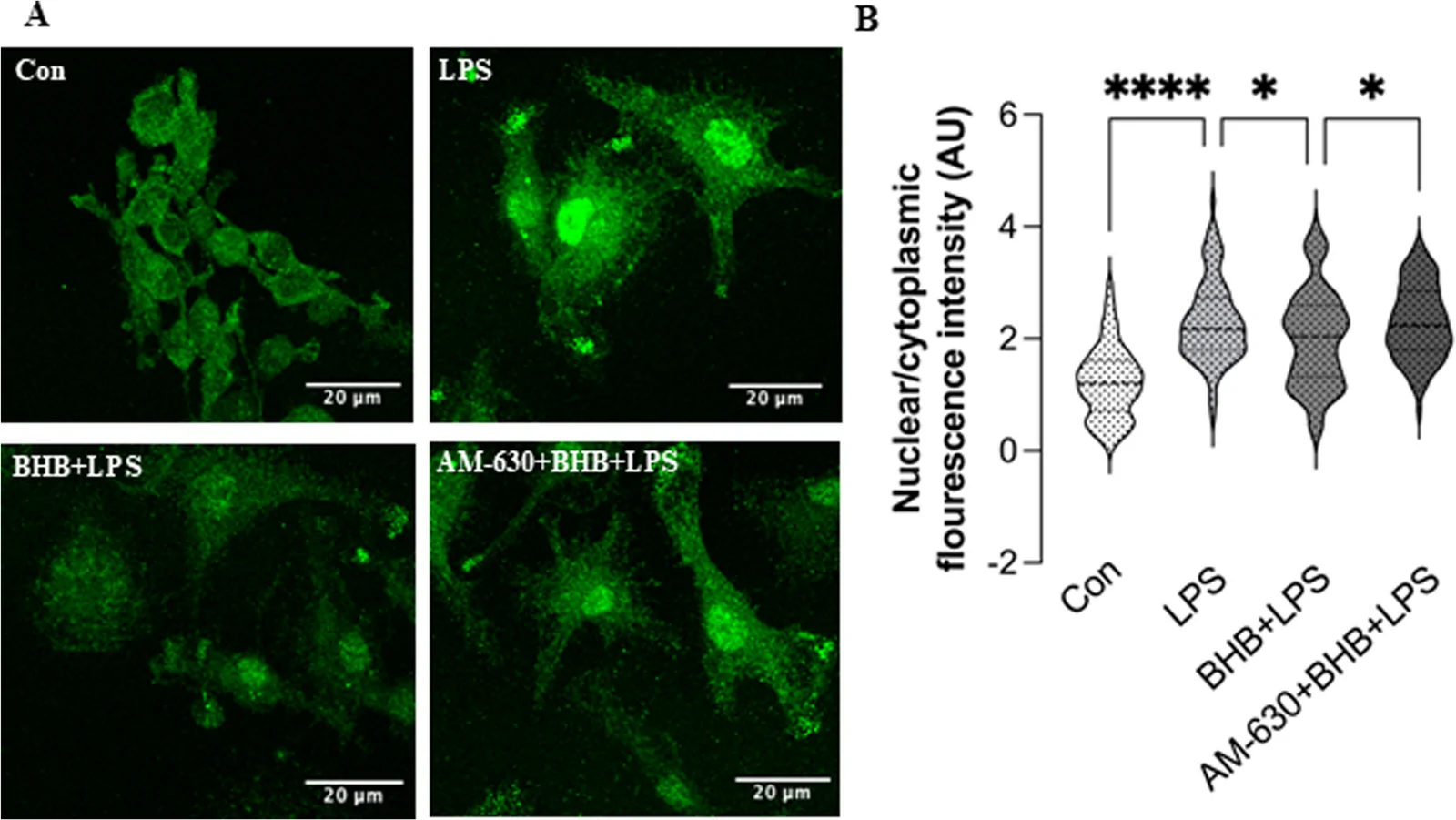
Inhibition of NF-κB nuclear translocation by BHB is partially reversed by CB2R blockade (**A**) Representative fluorescence images of primary mouse microglia stained with an antibody against NF-κB p65 (green). (**B**) Violin plot showing the nuclear-to-cytoplasmic intensity ratio of NF-κB p65 across treatment groups. Con – Control group; LPS – LPS-treated group for 24 h (100 ng/mL); BHB + LPS – 1 h pretreatment with BHB before LPS; AM-630 + BHB + LPS – 1 h pretreatment with AM630 (1 µM) before BHB and LPS. *n* = 90. Data are presented as mean ± SEM. **p < 0.01; **p < 0.0001

Next, we investigated whether BHB inhibits the NF-κB signaling pathway, a central regulator of microglial immune function that drives the production of pro-inflammatory cytokines and chemokines, thereby amplifying neuroinflammation. Additionally, we aimed to determine whether CB2R blockade could reverse BHB’s inhibitory effects on this pathway. To assess NF-κB activity, we performed a nuclear translocation assay. As expected, LPS stimulation significantly enhanced nuclear localization of NF-κB, increasing its translocation compared to control (Fig. 8A, B; *p* < 0.0001), confirming engagement of the inflammatory pathway. Pre-treatment with BHB prior to LPS exposure significantly attenuated this effect (Fig. 8A, B; *p* < 0.05), consistent with BHB’s reported anti-inflammatory properties. Notably, co-treatment with the CB2R antagonist AM-630 partially reversed the effect of BHB, increasing nuclear NF-κB localization compared to the BHB + LPS group (Fig. 8A, B; *p* < 0.05).

These findings suggest that BHB exerts its anti-inflammatory effects, at least in part, by suppressing NF-κB activation, a key transcription factor in microglial-mediated neuroinflammation.

## Discussion

BHB has been shown to improve memory, enhance cognitive function, and promote neuronal plasticity in various disease models, primarily through its anti-inflammatory effects [14]. However, the precise mechanisms underlying these effects remain unclear. In this study, we demonstrate that the anti-inflammatory actions of BHB are mediated, at least in part, through CB2R signaling.

We initially hypothesized that BHB exerts anti-inflammatory effects in the DIO mouse model, which is well established to develop low-grade neuroinflammation [40–42], characterized by reactive microglial cells and oxidative stress [41]. In the DIO model, BHB supplementation has been shown to reduce body weight and improve metabolic parameters [43–44]. Furthermore, BHB is widely recognized for its anti-inflammatory properties in aging mice and in neurodegenerative disease models. For instance, a ketogenic diet has been shown to attenuate age-related chronic neuroinflammation in mice [45]. Additionally, BHB administration in Alzheimer’s (5xFAD) mice model significantly reduced amyloid-β plaque burden, suppressed microgliosis, and inhibited NLRP3 inflammasome activation [46]. However, its specific effects on microglia have not been thoroughly investigated. To assess microglial function in the brain, we focused on three key aspects: morphology, phagocytic capacity, and the ability to engulf synaptic structures. Prior research has demonstrated that BHB treatment promotes a more branched microglial morphology, which is typically associated with a less pro-inflammatory, more surveillant state [47]. Additionally, Obesity is linked to microglial dysfunction related to microglial reactivity, which has been implicated in cognitive decline [48]. In line with this, we observed a transition from the rounded, amoeboid morphology typical of reactive microglia to a more complex, ramified structure in BHB-treated obese mice, indicating a shift toward a homeostatic, surveying phenotype. Moreover, BHB treatment appeared to shift microglia toward a more selective phagocytic profile, as indicated by increased CD68/Iba-1 colocalization together with reduced PSD95/Iba-1 colocalization. This suggests that BHB may enhance microglial phagocytic/lysosomal activity without promoting excessive engulfment of postsynaptic elements. Such selective modulation may reflect the functional reprogramming of microglia toward a more protective phenotype under metabolic stress.

Next, we aimed to investigate whether the immunomodulatory effects of BHB are accompanied by changes in components of the endocannabinoid system. CB2R is associated with immune modulation in the CNS, is highly expressed in microglia, and plays a pivotal role in regulating inflammatory responses [49]. Furthermore, MAGL is an enzyme that hydrolyzes the endocannabinoid 2-arachidonoylglycerol (2-AG) [50], an agonist of both CB1R and CB2R, into arachidonic acid and glycerol [51]. Inhibition of MAGL has been shown to elevate 2-AG levels, leading to neuroprotective effects in models of neurodegenerative diseases [52]. To our knowledge, no prior studies have directly linked the effects of BHB in the brain to the endocannabinoid system. However, a study by Gigante et al. reported that in a rat model of irritable bowel syndrome (IBS), a ketogenic diet restored epithelial integrity in the intestinal crypt base and was associated with upregulation of both CB1 and CB2 receptors [53], suggesting that BHB-related mechanisms may be modulated via the endocannabinoid system. In our study, we found that CB2R expression increased, whereas MAGL expression decreased, in the brains of obese mice treated with BHB. Together, these changes suggest that BHB treatment may influence endocannabinoid-related signaling in the obese brain. However, they do not directly demonstrate increased 2-AG levels or CB2R activation. HCA2 protein expression was also evaluated as a potential mediator of BHB signaling, given that BHB activates this receptor [18]. Hippocampal HCA2 expression was higher following BHB treatment, although the difference was not statistically significant. Nevertheless, this does not exclude its functional involvement in the observed effects of BHB.

Building on these findings, we extended our investigation to an in vitro model using primary mouse microglia. In our in vitro analysis, we found that BHB counteracted LPS’s detrimental effects by increasing phagocytosis and decreasing ROS production, consistent with prior observations of BHB’s involvement in phagocytosis and ROS production [17, 54–56]. Additionally, we demonstrated that the phagocytic capacity of microglia was significantly reduced when cells were pretreated with the inverse CB2R agonist AM-630, accompanied by increased ROS production. These findings suggest that BHB’s ability to enhance phagocytosis and reduce oxidative stress is dependent on functional CB2R signaling.

Moving forward, we aimed to identify key markers involved in the immunomodulatory effects of BHB and to determine whether CB2R blockade influences their expression. We observed a pronounced effect on *Arg1* mRNA levels, which were significantly upregulated following BHB treatment after LPS stimulation. This upregulation was abolished upon pharmacological blockade of CB2R, indicating that the expression of this marker is at least partially dependent on CB2R signaling. However, since mRNA levels do not always directly correlate with protein quantity, we further assessed Arg1 protein expression by Western blotting. The results confirmed the qPCR findings, demonstrating elevated marker protein levels following BHB treatment and reduced levels when cells were treated with AM-630.

Arg1 is widely recognized as a marker of the anti-inflammatory microglial phenotype [57], supporting the idea that BHB mediates its anti-inflammatory effects by upregulating Arg1. Usually, when Arg1 is elevated, it competes with iNOS for their common substrate, L-arginine, thereby limiting substrate availability for iNOS-mediated nitric oxide (NO) production and contributing to the resolution of inflammation [58]. Both our data and prior studies have demonstrated that BHB exerts beneficial anti-inflammatory effects.

CB2R blockade with AM-630 has been shown to reduce Arg1 expression in various disease models [59], whereas CB2R agonism typically produces the opposite effect [60]. In our study, pre-treatment of microglia with AM-630 reduced Arg1 expression. These results suggest that the immunomodulatory effects of BHB are highly dependent on CB2R signaling. Regardless of the specific downstream mechanisms, our findings suggest that CB2R-associated signaling contributes to selected BHB-induced effects in primary microglia. However, because CB2R blockade only partially affected these responses, our data do not support complete CB2R dependency.

Finally, we demonstrated that BHB treatment following LPS stimulation significantly inhibited NF-κB signaling. This observation is consistent with previous findings [18, 61]. Additionally, CB2R blockade reversed this effect. CB2 receptor has been previously implicated in modulating the NF-κB pathway [62], and our results further support its involvement and reveal convergence of CB2R and BHB actions in the NF-κB signaling pathway.

Taken together, our results demonstrate that BHB has an immunomodulatory effect in both an in vivo DIO model and an in vitro model of primary microglial cells. The data support the involvement of CB2R-associated signaling in selected beneficial effects of BHB in primary microglia. The partial attenuation of BHB-induced responses by CB2R antagonism suggests that CB2R may participate in signaling pathways that overlap with, or modulate, BHB-driven anti-inflammatory effects.

### Limitations of the study

One limitation of the present study is the absence of a non-DIO control group. Although our findings show that BHB modulates inflammatory and microglial parameters in obese mice, the current experimental design does not allow us to determine whether these effects represent a restoration toward a lean/homeostatic state or treatment-specific changes within obese conditions. Therefore, the in vivo findings should be interpreted as BHB-induced changes relative to untreated DIO mice, rather than as evidence of complete normalization of microglial morphology, phagocytic activity, synaptic engagement, or inflammatory marker expression. Future studies including a non-DIO control group will be required to establish the extent to which BHB shifts microglial and inflammatory profiles toward a lean physiological baseline.

Another limitation is that CB2R involvement in vivo was assessed primarily by measuring expression levels. However, expression changes alone do not establish functional or mechanistic involvement of CB2R in mediating the effects of BHB. Therefore, these findings should be interpreted cautiously. Further in vivo studies using pharmacological CB2R blockade or genetic approaches will be needed to determine whether changes in CB2R expression directly contribute to BHB’s anti-inflammatory and microglia-modulating effects. The potential involvement of HCA2 represents an additional limitation of this study. Although its expression was assessed in the hippocampus, HCA2 activity was not examined using pharmacological or genetic approaches. Therefore, the present findings cannot distinguish CB2R-associated effects from those potentially mediated through HCA2 or other BHB-responsive pathways. Future studies employing HCA2 inhibition or genetic deletion should clarify its contribution and the possible interaction between HCA2- and CB2R-associated signaling in mediating the immunomodulatory effects of BHB.

## Conclusion

In conclusion, this study shows that BHB modulates microglial inflammatory responses in both DIO-associated neuroinflammation and LPS-stimulated primary microglia. The partial reversal of selected BHB-induced effects by CB2R blockade suggests that CB2R-associated signaling may contribute to these responses. However, because direct interaction between BHB and CB2R was not assessed and pharmacological controls, baseline comparisons, and inflammatory readouts were limited, these findings should be interpreted as evidence of CB2R involvement rather than direct CB2R activation. Overall, this work supports further investigation of BHB as a metabolic modulator of microglial function in neuroinflammatory and metabolic conditions.

## Supplementary Information

Below is the link to the electronic supplementary material.


Supplementary Material 1


## Data Availability

All data generated or analyzed during this study are included in this published article. Raw data will be available under reasonable request.

## Abbreviations

AD: Alzheimer’s disease
Arg1: Arginase 1
BHB: ß–hydroxybutyrate
BSA: bovine serum albumin
CB2R: Cannabinoid receptor type 2
DIO: Diet–induced obesity
ELISA: Enzyme–linked immunosorbent assay
HI FBS: Heat–inactivated fetal bovine serum
LPS: Lipopolysaccharide
NF: κB–Nuclear factor kappa–light–chain–enhancer of activated B cells
PFA: Paraformaldehyde
ROS: Reactive oxygen species
TNF: α–Tumor necrosis factor–α
TTBS: Tris–Tween buffered saline
