# Correction: Unveiling the role of CB2 receptor in beta-hydroxybutyrate mediated modulation of microglial anti-inflammatory phenotype

**DOI:** 10.1007/s13105-026-01222-x

**Published:** 2026-08-28

**Authors:** Monika Iešmantaitė, Liutauras Usonis, Kristupas Čeika, Egidijus Šimoliūnas, Mantas Liudvinaitis, Nerea Soto-Arroyo, Isabel Moreno-Indias, Francisco J Tinahones, Daiva Baltriukienė, Virginia Mela

**Affiliations:** 1 Institute of Biochemistry, Life Sciences Center, Vilnius University, Vilnius, Lithuania; 2 Department of Endocrinology and Nutrition, Instituto de Investigación Biomédica de Málaga y Plataforma en Nanomedicina–IBIMA Plataforma Bionand, Málaga, Spain; 3 Center for Biomedical Network Research in Physiopathology of Obesity and Nutrition (CIBEROBN), Instituto de Salud Carlos III, Madrid, Spain; 4 Department of Endocrinology and Nutrition, Hospital Universitario Virgen de la Victoria–IBIMA Plataforma Bionand, Málaga, Spain; 5 Department of Medicine and Dermatology, Facultad de Medicina, Universidad de Málaga, Málaga, Spain; 6 Department of Surgical specialties, biochemistry, and immunology, Universidad de Malaga, Campus de Teatinos s/n, Malaga, Málaga, 29010 Spain; 7 Department of Biological Models, Vilnius University Life Sciences Center, Saulėtekio av. 7, Vilnius, 10257 Lithuania

**Correction: Journal of Physiology and Biochemistry (2026) 82:65**

**https://doi.org/10.1007/s13105-026-01206-x**

The Original article has been corrected to update the article title since the published version's title appears cut and is missing half of the title. The correct article title should be "Unveiling the Role of CB2 Receptor in Beta-Hydroxybutyrate Mediated Modulation of Microglial Anti-inflammatory Phenotype"

