# Supplementary figures and images for: Unveiling the role of CB2 receptor in beta-hydroxybutyrate mediated modulation of
Source: J Physiol Biochem. 2026 Jul 9;82(1):65. doi: 10.1007/s13105-026-01206-x (PMC13350206; doi:10.1007/s13105-026-01206-x)

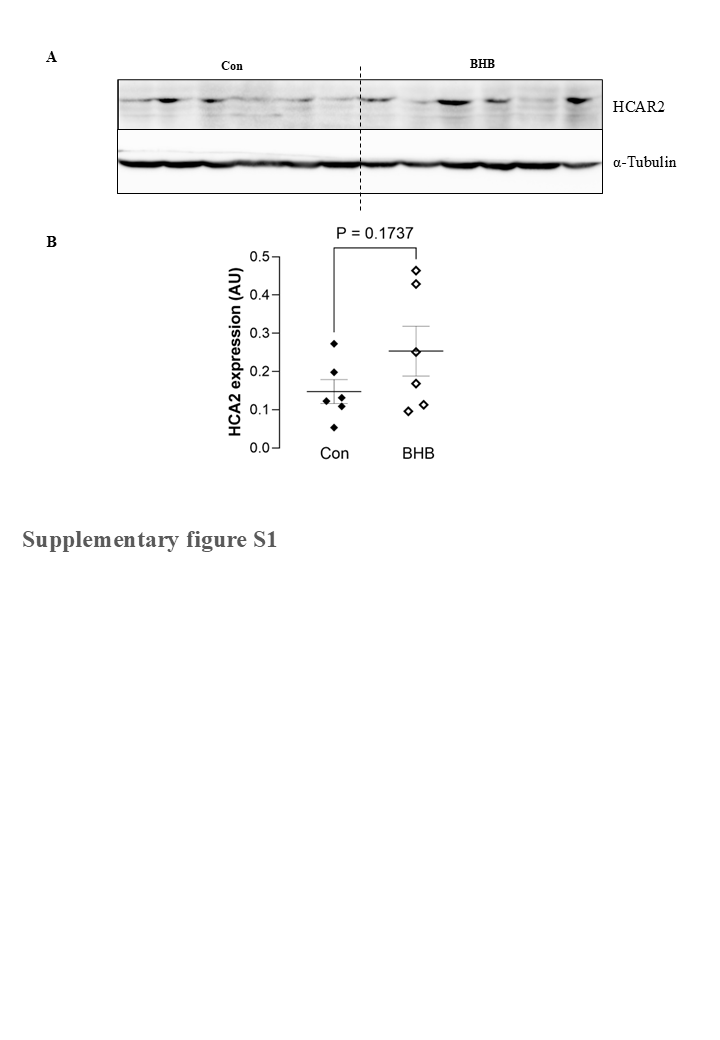

Supplement: Supplementary file 1 — Supplementary Material 1 [file 13105_2026_1206_MOESM1_ESM.tif]
